# Strong sustainability in coastal areas: a conceptual interpretation of SDG 14

**DOI:** 10.1007/s11625-017-0472-y

**Published:** 2017-09-07

**Authors:** Barbara Neumann, Konrad Ott, Richard Kenchington

**Affiliations:** 1 Department of Geography, Kiel University, Kiel, Germany; 2 Philosophical Seminar, Kiel University, Kiel, Germany; 3 Australian National Centre for Ocean Resources and Security (ANCORS), University of Wollongong, Wollongong, Australia

**Keywords:** Sustainable development goals (SDGs), Sustainability, Natural capital, Coastal zones, Coastal governance, Conservation

## Abstract

Humans derive many tangible and intangible benefits from coastal areas, providing essential components for social and economic development especially of less developed coastal states and island states. At the same time, growing human and environmental pressures in coastal areas have significant impacts on coastal systems, requiring urgent attention in many coastal areas globally. Sustainable development goal (SDG) 14 of the 2030 Agenda for Sustainable Development (henceforth the 2030 Agenda) aims for conservation and sustainable use of the oceans, seas, and marine resources, explicitly considering coastal areas in two of its targets (14.2 and 14.5). These promote, as we argue in this article, a strong sustainability concept by addressing protection, conservation, and management of coastal ecosystems and resources. The 2030 Agenda adopts the so-called “three-pillar-model” but does not specify how to balance the economic, social, and environmental dimensions in cases of trade-offs or conflicts. By analysing SDG 14 for the underlying sustainability concept, we derive decisive arguments for a strong sustainability concept and for the integration of constraint functions to avoid depletion of natural capital of coastal areas beyond safe minimum standards. In potential negotiations, targets 14.2 and 14.5 ought to serve as constraints to such depletion. However, such a rule-based framework has challenges and pitfalls which need to be addressed in the implementation and policy process. We discuss these for coastal areas in the context of SDG 14 and provide recommendations for coastal governance and for the process ahead.

## Introduction

Coastal areas are frontiers along roughly 356,000 km of global coastline (Central Intelligence Agency 2016). Located at the interface of land and sea they integrate marine and terrestrial processes through mutual interactions (Woodroffe 2002: 2), including anthropogenically driven land–sea interactions. They contain a variety of marine and terrestrial ecosystems covering the range from natural to highly altered environments. Coastal ecosystems such as mangroves, salt marshes, coral reefs, beaches, and dunes provide regulating ecosystem services (ES) such as protection from coastal hazards like storms and surges, coastal flooding, and erosion (Agardy et al. 2005; Martinez et al. 2011; Spalding et al. 2014). These habitats are also highly relevant in terms of provisioning ES, e.g. as nursery areas for maintenance of fish stocks in the case of mangroves (Brander et al. 2012). Natural resources from oceans and coasts are essential components for human well-being. The world’s coastal areas generate a large share of the ocean’s services, and their support of coastal economies and livelihoods is particularly important in less developed areas (Costanza 1999; Martínez et al. 2007; Kildow and McIlgorm 2010; Visbeck et al. 2014). But people are also drawn to the coast for recreational, aesthetic, cultural, and spiritual reasons, for the specific sense of place and well-being they attach to coastal environments (Bell et al. 2015), or for pursuing “coastal lifestyles” (Green 2010). For coastal states and island nations, coastal tourism is a complex factor for conservation and economic development (Kenchington 1993). For many Small Island Developing States (SIDS), unique land- and seascapes enable tourism as a major economic activity (Division for Sustainable Development 2015; UNEP 2009).

Coastal zones are attractive environments to settle and live or pursue economic activities, but this has also led to a growing human footprint on coastal ecosystems, including less charismatic but ecologically highly important ones like seagrass meadows or salt marshes, and become a threat to many species (Duarte et al. 2008; Stojanovic and Farmer 2013). Importantly, coastal zones sustain complex interactions of marine and terrestrial habitats supporting high biodiversity and complex life cycle and food chain linkages through the water column. The dynamics of most marine ecological linkages are poorly understood compared to terrestrial ecosystems.

The main threats to coastal ecosystems are described as habitat loss or conversion due to coastal development, agriculture, or aquaculture; habitat degradation due to eutrophication, pollution, and contamination; and consequent changes in sediment and water supply due to human activities along the coasts and in the upstream watersheds (Agardy et al. 2005: 539; Newton et al. 2012). Further pressures arise from climate change, invasive species, and overexploitation of fishing resources. Coastal zones are typically subject to natural hazards such as river flooding, storms and storm surges, and tsunamis, with serious socio-economic impacts from flooding and erosion in developed coastal areas (Newton and Weichselgartner 2014). Some of these effects are exacerbated by climate change and sea-level rise (Wong et al. 2014), and projected increases in the frequency and intensity of natural hazards as well as preventative measure taken to protect coastal property are further causes of habitat degradation.

Population growth, urbanisation trends and increasing demand and competition for resources, transport, and energy are placing growing pressures on coastal zones, their ecosystems, and the capacity to produce sustainable resources (Neumann et al. 2015; Merkens et al. 2016; Agardy et al. 2005). Meanwhile, poor planning and incoherent and fragmented land–sea governance, and a lack of awareness, regulations and enforcement are adding to the problems (Duxbury and Dickinson 2007; Visbeck et al. 2014). The resulting changes affect human well-being directly and indirectly through many interlinkages and causal relationships, though not necessarily in a linear, one-directional or universal way (Crossland et al. 2005: 15–17; Sekovski et al. 2012; Pinto et al. 2014).

Generally, the ocean and its coasts are expected to create new economic opportunities and substantial growth in the marine and maritime sector in both developed and developing countries (European Environment Agency 2013: 12–15; Ehlers 2016). Europe has adopted an explicit strategy targeting “blue growth” (European Commission 2012). SIDS are also placing great hopes on a “blue economy”, a term developed from the “green economy” approach to sustainable development and poverty eradication by various governmental and non-governmental actors in advance of, and presented at, the United Nations’ Rio+20 Conference on Sustainable Development in 2012 (UNDESA 2011; United Nations 2012; Silver et al. 2015). Considering the multiple environmental, social, and economic impacts from increasing resource use and development, the formulation of principles and guidelines on how to ensure a sustained provision of the services delivered by oceans, seas and coastal areas is extremely relevant under any concept of blue growth. Coastal tourism, fisheries and the many other aspects of coastal economies and livelihoods rely strongly on “healthy” coastal ecosystems for a sustained provisioning of the desired services (Agardy et al. 2005; UNEP 2009; Division for Sustainable Development 2015).

The Rio+20 outcome document *The Future We Want* acknowledges the critical role of “oceans, seas and coastal areas” in sustaining the “Earth’s ecosystem”, and emphasises the need for “conservation and sustainable use of the oceans and seas and of their resources” (United Nations 2012: section 158). It commits to “protect, and restore, the health, productivity and resilience of oceans and marine ecosystems” by effectively applying “an ecosystem approach and the precautionary approach in the management […] of activities having an impact on the marine environment, to deliver on all three dimensions of sustainable development” (United Nations 2012: section 158). The *2030 Agenda for Sustainable Development* (henceforth the 2030 Agenda) commits to these aspirations through a specific sustainable development goal (SDG) on the conservation and sustainable use of the oceans, seas and marine resources (SDG 14) among the newly established 17 SDGs (United Nations 2015). SDG 14 explicitly addresses coastal areas and ecosystems in two of its main targets (14.2^1^ and 14.5^2^). Further targets under SDG 14 as well as targets under other goals, though not explicitly referring to coastal areas, are implicitly relevant for coastal areas and for the protection, conservation and management of coastal ecosystems and resources.

In this article, we address questions of interpreting and implementing the principles and guidelines set out under SDG 14 to meet the challenges faced by coastal areas. We analyse SDG 14 for its underlying sustainability concept, concentrating our analysis on the targets that explicitly address coastal areas (14.2 and 14.5). We derive decisive arguments for a strong sustainability concept and for the integration of constraint functions to avoid depletion of natural capital of coastal areas beyond safe minimum standards. We further discuss obstacles to applying strong sustainability to coastal zones and how these might be addressed in the policy development and implementation processes, and draw conclusions for the way ahead. Since “sustainability” is essentially a normative concept, our analysis and interpretation of SDG 14 targets 14.2 and 14.5, and of the challenges and pitfalls of implementing SDG 14, is developed through immersive ethical discourse analysis.

To set the stage, it seems appropriate to provide a working definition of “coastal zone” or “coastal area”. Coastal zone definitions may be based on governmental or legislative boundaries (cf. Sekovski et al. 2012), employ distance measures or topographical classifications (cf. Neumann et al. 2015), refer to biophysical characteristics of coastal landscapes or ecosystems, or present combinations of these (cf. Agardy et al. 2005; HORSCERA 1991). However, many processes driving coastal challenges originate and play out beyond biophysical or administrative boundaries (Duxbury and Dickinson 2007). Thus, a broad catchment-to-ocean definition as the “Margin” used by Glavovic et al. (2015) seems most appropriate, but difficult to operationalise. The Commonwealth of Australia acknowledged this in their coastal policy by considering “the boundaries of the coastal zone […] to extend as far inland and as far seaward as necessary to achieve the Coastal Policy objectives, with a primary focus on the land–sea interface”; however, noting that the policy could not deal with “all issues associated with catchment and marine management” even though coastal zones, in their definition, contain terrestrial and marine areas (Commonwealth of Australia 1995). The targets under SDG that explicitly mention “coastal ecosystems” (14.5) and coastal areas (14.5) provide no clear delimitation or demarcation of the area that they refer to, and neither does SDG15 which deals with “terrestrial ecosystems”. Arguing that coastal systems include transitional systems which, in many cases, cannot be divided in either land or sea, we, therefore, define coastal zones as the area, water column and seafloor between the landward extent of tidal influences, including typical coastal ecosystems at the land–sea interface (e.g. dunes, coastal wetlands and lagoon systems), and the 12-nm territorial waters boundary or the 100 m depth contour, assuming that this delimitation encompasses most of the action and impacts. This definition is not intended to exclude areas with heavily modified or degraded coastal ecosystems. We concede that this and any other definition entails fuzziness and might be adapted to specific circumstances.

## The argument for a strong sustainable SDG 14

The 2030 Agenda and the SDGs are designed to be more inclusive, coherent and universal than previous declarations and the Millennium Development Goals (MDGs) (Martens and Obenland 2016). Environmentally oriented goals on water (SDG 8), climate (SDG 3), ocean and coasts (SDG 14), and terrestrial ecosystems (SDG 15) have been integrated to make the SDGs more comprehensive than their predecessors (United Nations 2015). With some optimism, we regard the SDG process as unprecedented opportunity to reconcile competing sustainability concepts by commonly shared endeavours to come close to (or even reach) the SDG-objectives. The SDGs seek to “balance all three dimensions of sustainable development: the economic, social and environmental” (United Nations 2015). In this statement, the so-called “three-pillar-model” has been implicitly adopted. This model is highly attractive for policy makers because many different political programmes can be said to be in accordance with the three-pillar-model. The three-pillar-model leaves open, though, how balancing should be done in hard cases of trade-offs, conflicts, risks, and uncertainty. It has been accused that its application often downplays the ecological dimension because economic goals and social needs may appear to be more urgent.^3^

Facing these challenges, we hold that all SDG-targets focussing on natural systems, as SDG 14, should explicitly rely on the concept of strong sustainability. This section outlines an argument in favour of this prescriptive claim proposing a complex normative framework for coastal governance to meet the requirements of SDG 14. Our argument partly points at presuppositions of SDG 14, and it partly interprets SDG 14 in favour of strong sustainability. A constitutive principle of “strong” sustainability requires keeping the (critical) substances of all natural capitals constant over time (constancy of natural capital rule, CNCR) irrespective of how other stocks of societal capitals evolve (Daly 1996). This principle clearly should be both substantiated and interpreted with respect to the crucial concepts being involved, as “critical substance”, “constancy”, and “time scales”. The arguments in favour of strong sustainability should meet the requirement of broad acceptance within the overall SDG process, of epistemic communities, local stakeholders, and policy makers. In making these arguments, we identify five conceptual and methodical aspects to be interpreted and analysed:

Different concepts of sustainability should be made explicit and weighed in the context of the SDGs (1); the presupposition of a 10% objective as formulated in target 14.5 should be understood (2); a scientific meaning of metaphors (e.g. “health”) used in conjunction with environmental entities and domains in the 2030 Agenda and SDG 14 should be established through interpretation, here regarding target 14.2 (3); the method and role of the ES-approach within SDG 14 should be analysed (4); and, finally, a conceptual scheme for assessing and judging conflicts and trade-offs should be given (5).

Each aspect is addressed in the following subsections. Taken together, this argumentative framework may serve for orientation and further discourse on SDG 14 and, more specifically, coastal governance.

### Concepts of sustainability and the SDGs

Broadly speaking, there are three competing theoretical approaches of how to conceive the ethical idea of sustainability: Through (1) need-based or capability-based humanitarian approaches, (2) economic approaches based on welfarism, and (3) natural resource-based, strong sustainability approaches.

#### Humanitarian approaches to sustainability

There are theories of sustainability which directly and foremost address pressing humanitarian challenges, as absolute poverty, food and freshwater insecurity, deficits in access to health care, education, and the like. These approaches have been substantiated by the report “Our Common Future” of the World Commission on Environment and Development (World Commission on Environment and Development 1987). The WCED’s famous definition of sustainable development entails the concept of basic human needs being enlarged to intergenerational equity: meeting the needs of the present should not compromise or impair the fulfilment of future needs. This need-based-definition was adopted because the basic needs of the poor served as common moral denominator of this UN-commission. WCED does not assume absolute ecological limits but rather sees restrictions in the societal and technological capacity to utilise nature (WCED 1987: 43), in contrast to contemporary discussions that consider “planetary boundaries” and a “safe and just operating space” for humanity in the context of sustainable development and the SDGs (Rockström et al. 2009; Griggs et al. 2013; Hajer et al. 2015; Steffen et al. 2015). The WCED report is a significant document because the Rio Summit (UN 1992) largely relied on this report. In some sense, the SDGs are the legacy of the WCED.

The humanitarian sustainability-approach has been recently theorised via the so-called “capability approach” (CA) (Sen 1999, 2005; Nussbaum 2003, 2006; Robeyns 2005; for critical debate see Ott 2014). The CA-approach is grounded in a theory of justice (Sen 2009). According to Sen, the currency of justice should be human capabilities. Sen accuses theories of justice that focus on resources of committing the fallacy of “resource fetishism”. To Sen, resources are means only, while human capabilities (beings and doings) are ends in themselves. Therefore, the natural environment belongs to the circumstances of justice only. According to Nussbaum, each human being is entitled to live a dignified life. Such life requires the performance of specific capabilities. Nussbaum (2003) presents a list of ten capabilities. In her view, injustice always occurs if individuals must live beyond a specific threshold of each single capability. Therefore, the amount of global injustice becomes strongly dependent on stipulations of capability thresholds (Ott 2014). Among these ten capabilities, there is the capability of being able “to live with concern for and in relation to animals, plants, and the world of nature” (Nussbaum 2003: 42). However, neither “concern” nor “relation” is further specified.

Implicitly endorsing the capability-approach, the SDGs focus largely on humanitarian aspirations. As a result, 14 out of 17 goals refer to societal objectives. It comes as little surprise that the concept of planetary boundaries (Rockström et al. 2009; Steffen et al. 2015) has not been integrated into the SDG-declarations and that the normative core of the 2030 Agenda is to ensure that “no one will be left behind” (United Nations 2015).

#### Economic approaches to sustainability

Economic sustainability-approaches, which have a theoretical background in economic welfarism, operate under the idea that, on the aggregate, human utility should not decline over time. They have been theorised by Heal (1998), Chichilnisky (1997), and Asheim (2007), and they have been operationalised through the genuine-savings-measure (Atkinson et al. 1997). This approach perceives any kind of capital as being an asset within a portfolio. The benevolent decision-maker takes the role of an ideal (utilitarian) portfolio manager who wishes to maximise societal welfare. Since a specific virtue ethic underlies economic theory (Hodgson 2001: 60–63), the portfolio manager decides under the discount rate maxim not to waste scarce factors of production. Here, in principle, nature conservation is a wasteful activity if conversion of natural systems could create more immediate (discounted) overall welfare (=net present value). A paradigm case might be to convert a natural coastal area into a harbour area, urban suburb, aquaculture facility, or tourist destination.

In principle, any single asset of the portfolio can be substituted by another one if the latter yields higher welfare. Thus, the portfolio manager is entitled to substitute stocks of natural capital with man-made capital or human capital (e.g. education). If the degree of substitutability is assumed to be high or very high, as Solow (1974) guessed, the loss of natural capital is neither horrible nor repugnant if investments in other kinds of capital are properly undertaken. This approach has been dubbed “weak sustainability”.^4^

Within this approach there are reasons to protect nature only if nature is understood as “critical” for human welfare, or if persons have strong preferences for conservation (so-called “existence value”). Quite often economic approaches adopt a “safe minimum standard” (SMS) of natural assets out of a sense of precaution (Hampicke 1992: 310–314). If so, one may ask how safe might be safe enough in the longer run. The more environmentally risk averse a decision maker is, the more natural assets should be preserved, conserved, or even restored. A highly risk-averse SMS could come close to a CNCR.

The portfolio manager must also respect the preferences of consumers. Strictly speaking, economists themselves do not value but seek to allocate scarce resources in such ways as to maximise the fulfilment of given preferences. If many humans have preferences to see natural environments being protected since they enjoy encountering unspoiled nature, bird-watching, etc., economists must respect the values of such kinds of pleasures. If people value the mere existence of natural beings (e.g. natural monuments as the Great Barrier Reef, rare species as blue whales, or lonely tropical beaches), economists are committed to hold such assets in the portfolio. Conceptual schemes as the Total Economic Value (TEV) entail option values, bequest values, and existence values (Pearce and Moran 1994). Indirect methods such as contingent valuation, travel cost analysis or choice experiments are being used to measure such preferences in monetary terms. If people would value a walk along a natural coast or days on remote beaches as high or greater than say, a shopping weekend in a city, it would be perfectly reasonable, economically, to protect and restore many coastal zones. This also holds if living close to the beach is, as in Australia, part of a cultural lifestyle which many people wish to maintain (Gurran and Blakely 2007; Green 2010).

We conclude that, within economic approaches to sustainability, there is a tension between a growth-theoretical, high discount rate portfolio approach which assumes high degrees of substitutability between assets, and approaches which adopt SMS and take preferences in favour of nature conservation more seriously. While the former remains within the theoretical scope of weak sustainability, the latter tend, via TEV and SMS, gradually toward strong sustainability.

#### Natural resource-based approaches and strong sustainability

Further, there are concepts that directly focus different critical stocks of natural capital from an intergenerational perspective. These concepts have deep roots in history (von Carlowitz 1713; Jefferson 1789, Möbius 1877). Thomas Jefferson’s famous dictum “that the Earth belongs in usufruct to the living” (Jefferson 1789) builds on the continental tradition of *usufruct* (German: “Nießbrauch”). *Usufruct* constrains legitimate ownership because the “substance” of a specific good must be maintained over time, be it a castle, a vineyard, fertile soils, or forests. *Usufruct* can be applied, in principle, both to private property and to commons. This resource-based concept of sustainability has been applied to oyster banks by Möbius (1877), and it was dominant until the years before First World War (Ott 2008).

In recent times, this more “resourcist” approach has been theorised by Daly (1996), Ekins et al. (2003), and Ott and Döring (2011) as “strong sustainability”. It relies on the pre-analytical vision that humans must learn to live within limits on a bountiful but finite planet. From an ethical perspective, this concept has two moral building blocks: (1) environmental ethics, and (2) theories of inter- and intra-generational distributive justice. While environmental ethics make explicit all the different values which can and should be attributed to different natural entities, distributive justice argues for specific schemes of how to distribute natural goods fairly among claimants and among different generations under conditions of moderate scarcity. Environmental ethics constitutes natural beings as (precious and often fragile) valuable goods (resources, funds, stocks), while distributive justice asks how these goods should be distributed within space and time. The concept of strong sustainability supposes that many natural goods have a rather low degree of substitutability, may increase in value over time, are of cultural (*eudemonic*) significance (Ott 2016) and may mean even more to future persons than to contemporary ones, and that they deliver a multitude of “services” (see “The methodical approach of “ecosystem services””).

Strong sustainability provides several lines (patterns) of arguments why the remaining (critical) stocks of natural capital in their heterogeneity and diversity (such as forests, grasslands, lagoons, dunes, salt marshes, etc.) should be maintained and preserved out of long-term precaution and prudence. Arguments in favour of strong sustainability are provided by Daly (1996), Neumayer (1999), Ott (2011; 2014) and Ziegler and Ott (2011). Strong sustainability can be operationalised via a “kinetic” concept of resources, stocks, and funds (Klauer et al. 2016, in German: “Beständeperspektive”). Pollutants are stocks which should decrease over time, while living funds as fish or mangroves should increase. It is crucial for strong sustainability not to treat living funds as stocks. Thus, the term “fish stocks” is, strictly speaking, misleading because fish are living funds (Ott and Döring 2011: 261 ff.).

With respect to justice, Ott (2014) argues that strong sustainability can be reasoned within Rawls’ famous Theory of Justice (Rawls 1971) as, in Rawls’ terms, a fair saving schedule that holds between generations. Leaving some philosophical details aside, this fair saving schedule can be conceived as fair bequest package if natural capital is recognised as being a real kind of capital. The details of such a bequest package cannot be fixed in the original position (“behind the veil of ignorance”) but must be determined within the branch of a well-ordered society which deals with collective goods (Rawls 1971: §43). Citizens of well-ordered democratic societies may support nature conservation policies out of different motives and traditions (Sagoff 1988: 137–156). In this perspective, sustainability policies are, primarily, anchored at the level of the national state, but they can be tightened to local communities or widened to international regimes, as in the ongoing SDG process. To Rawls, however, national states are organised in ways that enable them to protect collective legacies by means of law.

Strong sustainability is a rule-based concept. Under the general and supreme rule to hold natural capital constant over time (CNCR), there are several more specific management rules which apply to non-renewable resources, non-living and living funds. Another rule refers to past errors and failures. If too much natural capital has been consumed in the past, and the original substance has been diminished, people are committed, where possible, to restore natural capitals. What might, from an economic perspective, be perceived as investments in natural capital, may, from an ecological perspective, be seen as restoration and rehabilitating activities. Target 14.2 speaks of recovery and restoration which is clearly in line with this “corrective” dimension of strong sustainability. In the case of coastal zones, improvement of coastal water quality, reforestation of mangroves, or designation of protected areas for marine biodiversity may be regarded as such investments in living funds which provide different services to humans. If reduction of fisheries effort is a prerequisite for re-growth of the living funds of fishes, reductions of quotas may count as prudent investments, even if they involve heavy social and political costs. The “constancy” of the CNCR must be interpreted in a way that opens some leeway of choice at local scales. The substance of a coastal zone should be held constant even if, for example, the species composition may change over time. If salt marshes have been drained irreversibly, other types of wetlands might substitute them.

Rules of strong sustainability must be operationalised as political objectives (goals, targets) to guide policy decisions and implementation. Objectives can hold for different spatial scales. The transformation from rules to objectives may bring a spark of arbitrariness in the concept of strong sustainability because quantified targets (=objectives) cannot be logically derived from rules. Nevertheless, targets are necessary for environmental policy making. If a society supports ambitious objectives, there is a reasonable presumption that it wishes to follow the rules. Thus, one can infer backward from objectives being adopted to the underlying concept of sustainability.

### The presupposition of a 10% conservation objective

SDG-target 14.5 defines a global objective by aiming at conserving “at least 10 per cent of coastal and marine areas” by 2020 (United Nations 2015). The 10% conservation target was adopted in 2004 by the Conference of Parties (COP) to the Convention on Biodiversity (CBD 2004: decision VII/30, Annex II) and reaffirmed at the 10th CBD COP in 2010 as AICHI Biodiversity Target 11 (CBD 2010a). In our reading, target 14.5 implicitly comprises a 10%-target for marine protected areas and a 10%-target for protected coastal zones. Furthermore, SDG target 14.5 implicitly says that mankind should better reach out for more than 10% in the longer run. This 10%-objective as such is silent on the distribution of these protective areas along coastal zones globally. It is also silent under which conservation category specific coastal areas are to be protected, as discussed in “The 10% conservation target”.

In 2010, the CBD noted that “some […] 5 per cent of coastal areas are protected” (CBD 2010b). Since then, several large marine protected areas have been established (Hilborn 2016), but mostly in remote and offshore areas. Recommendations for larger percentages have been made by conservation scientists (Hilborn 2016; O’Leary et al. 2016), but scientists also remind us of the relevance of further aspects such as locality, protection status or management measures for ensuring positive conservation outcomes (Edgar et al. 2014; Devillers et al. 2015; Jones and De Santo 2016; Agardy et al. 2016). Thus, the 10%-target should be a baseline, not a ceiling. However, aiming to reach this target would mean doubling the coastal protected areas within few years.

Whoever adopts the 14.5 target has implicitly endorsed a principle or rule to protect (critical) natural capital. If a goal *g* is reasonable if, and only if, a principle *p* is endorsed, a commitment to *g* pragmatically implies a commitment to *p* (Ott 1997). If so, strong sustainability is implicitly present in SDG 14 as a kind of underlying presupposition. This “argument from presupposition” is an essential building block for our overall argument.

### The metaphorical vision of “healthy” ecosystems and “healthy ocean”

The 2030 Agenda and SDG 14 speak about a “healthy environment” (preamble) and a “healthy and productive ocean” (target 14.2) (United Nations 2015). Since the concept of health, whatever its meaning is within medicine, applies primarily to organisms rather than to multi-species interdependent ecological systems, it cannot be expanded so far as to be attributed to the physical entity named “ocean”. If so, the vision of a “healthy ocean" is stricto sensu just a metaphor (Rapport 1995) and, as such, a guiding image (“vision”). They can inspire our common moral imagination of how things should be (Weston 2012) and may motivate people more strongly than sophisticated ethical arguments. Such metaphors are “boundary objects” in sociology of science since they interconnect epistemic communities (as marine scientists and ecologists) and play a role at the intersection of science and politics. They can serve as such boundary objects if, and only if, they are translated into the concepts being used in these sciences. Otherwise they have some ideological and persuasive smell.

The “healthy ocean”-metaphor stands for a set of states of affair at the sea and even at coastal zones which (most or almost all) (prudent) humans wish to reach, wish to maintain, or wish to regain. Such states are often associated with scientific ecological concepts such as productivity, fertility, biological diversity, abundance, resilience, or function, which translate the health-metaphor into scientific discourse. A range of recent scientific research employs the term “ocean health” in such contexts, above all the widely applied and revisited “ocean health index” which was first presented by Halpern et al. (2012) (see also Halpern et al. 2015; Lowndes et al. 2015).

A paradigm case of such translation of “health”-ideas is to be found in Aldo Leopold’s environmental ethics (Leopold 1949). Leopold himself was influenced by the superorganism-concept in ecology as being proposed by Clements (Clements 1916, in Golley 1993). Although this concept was sharply rejected by Tansley (1935), Leopold never dropped it completely since he held strong pre-analytical intuitions and visions about “healthy” and “sick” land. In his famous principle, Leopold states that all actions which have impacts on land-use systems should respect “stability, integrity, and beauty” of the land.^5^ This principle has had a career in ethical ecocentrism (Callicott 1980; Westra 1994), while we wish to interpret it as a more pragmatic maxim of how to deal with land and sea, and more specifically with coastal areas (Norton 2013).

We offer the following interpretation: the concept of “stability” has lost credit in ecology and has been replaced by the resilience concept (Grimm and Wissel 1997; Chapin III et al. 2009). Though the concept of “integrity” has been taken up by science and in environmental management, and found wide usage in the context of ecosystem service assessments (e.g. de Groot et al. 2000; Burkhard et al. 2012), it remains contested. “Integrity” often supposes ideas of equilibria which are contested in modern ecology (Botkin 1990; Potthast 1999). The most promising strategy to specify “integrity” might be to point at (projective dispositional) concepts like productivity and fertility (Rolston 1988). “Integrity” does not refer to a specific state of a system but to the capability of living systems to produce and maintain complex ordered (*neg*-*entropic*) structures. The concept of “beauty” not only refers to aesthetic experiences of humans, but also to the overall richness and diversity of different biomes which might be appreciated as a “bounty” by humans. If conceived this way, “beauty” refers to all cultural values of nature (see “The methodical approach of “ecosystem services””).

To sum up, we propose to keep the concept of a “healthy ocean” as an inspiring metaphor, translating it in a (pragmatised) Leopoldinian spirit as “resilience, fertility, and cultural richness” of natural systems which should be maintained. Thus, the SDG-parlance (“health”, “resilience”, and “restoration”) only makes sense if a rather strong version of sustainability has been presupposed.

### The methodical approach of “ecosystem services”

The original idea of the ES approach, which was brought to broader recognition by Costanza et al. (1997) and Daily (1997), and was further advanced through the Millennium Ecosystem Assessment (2003, 2005), was to bridge the gap and recognise the interdependencies between nature and human welfare.^6^ Further, ES also are a device to specify the concept of natural capital. Parts of nature are being conceived as a rich and fertile set of stocks and funds (“capital”) that yield ongoing flows by which humans are benefitted in many ways. Such flows are dubbed “services” (although nature should not be perceived as service industry). Not all of nature is beneficial to humans, and there are many natural disservices as pests, tsunamis, or tropical cyclones. Such disservices and disasters may increase via climate change, marine pollution, and ocean acidification, but we will leave them aside and focus on positive services.

Four large kinds of human-related ES are to be distinguished: supporting, providing, regulating, and cultural services. It is contested whether the supporting services are to be regarded as a distinct category. For the sake of parsimony and to avoid double-counting, we leave supporting services aside. There are strong parallels between these different kinds of services and categories of values being reflected in environmental ethics: provisioning and regulating services refer to reliance and instrumental values, while cultural services mainly refer to so-called *eudemonistic* values that are oriented in the direction of “good life” and spiritual or personal well-being (Ott 2016). The (often underrated) domain of cultural services entails not only aesthetics or scenic beauty, leisure, recreation, inspiration, education and knowledge, and spiritual encounters with nature, but also cultural heritage (de Groot 2011; Barbier et al. 2011). Different services can be measured either in non-monetary (biophysical, social) or monetary terms (Braat et al. 2014: 35–41). It is widely agreed that cultural values are hard to monetise but are of paramount significance to many people (Chan et al. 2012; Jax et al. 2013).

The domain of cultural values should not be marginalised with respect to coastal zones since many humans have strong emotions, affiliations, and bonding to the sea and its coastlines (for examples see Kelly and Hosking 2008; Ratter and Gee 2012). The ES approach can and should be inclusive of non-Western cultural values which might be encapsulated in narratives, proverbs, songs, festivals, and rituals, etc. It can reach a deeper understanding of cultural ways of life on islands, coastal lands and waters including the meaningfulness in living a “coastal” life. Cultural values are present also in coastal activities which are undertaken for their own sake, such as sailing, swimming or diving, even if performed by visitors and tourists. Such meaningfulness might be downplayed in homogenising concepts of welfare, development, and even poverty eradication.

Nature can either be protected *de dicto* or *de re* (O’Neill 2015). If it is protected *de dicto*, one can replace a forest by another one which brings about the same services. Here, substitution re-enters nature conservation, making parts of nature more fungible to management considerations. If parts of nature are to be protected *de re*, a site counts as valuable in its uniqueness. The Wadden Sea, the Great Barrier Reef or the Galápagos Islands are famous examples for *de-re*-sites, but there are many less famous ones all around the world. Whether a site is a *de-re*-site or not depends on public deliberation. If one assumes that there are many *de-re*-sites at coastal zones, one has committed oneself to strong sustainability because *de-re*-sites are beyond the range of substitutability. The more *de-re*-sites, the stronger concept of sustainability will be.

Thus, from a purely methodological point of view, the ES approach remains neutral against competing concepts of sustainability and SDG-targets. If, however, one takes the domain of cultural values and the* de-re*-topic seriously, this substantial interpretation of ES justifies rather strong sustainability.

### Conceptual scheme for assessing and judging conflicts and trade-offs

Common denominators as the 2030 Agenda with its 17 SDGs and 169 targets, (strong) sustainability, or the ES approach should not blind us against the many tensions, trade-offs, and conflicts that occur under non-ideal conditions of policy development, planning, and implementation. Some initial attempts have been made to identify, analyse, and quantify the logical relations that hold between the different SDGs and their targets, deriving recommendations for dealing with these interactions (Nilsson et al. 2016; Stafford-Smith et al. 2016; Le Blanc et al. 2017; Singh et al. 2017; Schmidt et al. 2017). Goals and targets may be mutually supportive (“win–win”) or even indispensable, they may be neutral against each other, they may conflict with each other, or they may be incompatible. Interlinkages and interdependencies may be one- or bidirectional, show different levels of strength, and occur between policy sectors (as displayed by the different goals) or even between different jurisdictions (Nilsson et al. 2016; Stafford-Smith et al. 2016; Griggs et al. 2017). This is a complex setting for a successful implementation of the SDGs.

For the context of ocean and coasts, Schmidt et al. (2017) provide a comprehensive first-order assessment of key interlinkages between SDG 14 and other goals and targets under the 2030 Agenda. For the coastal targets 14.2 and 14.5, Schmidt et al. (2017) identified strong positive, reinforcing and often bi-directional interlinkages with the goals addressing poverty (SDG1) and hunger (SDG2), sustainable economic growth (SDG8), settlements (SDG11), and climate change (SDG13). But they also found negative interactions, for example constraints for poverty eradication or for sustainable and resilient housing and infrastructure, that could arise from marine protected areas when restricting access, limiting options or creating competition for scarce resources (Schmidt et al. 2017). This mirrors the critical role that coastal areas play for human well-being as well as the critical role that human interventions and actions play for the “health” and safeguarding of coastal natural capital. Considering the growing pressures on coastal areas due to population growth, urbanisation and intensification of resource utilisation, achieving sustainable development along all dimensions will require dealing with such trade-offs and conflicts.

With high probability, the unresolved theoretical contest between competing sustainability approaches will re-emerge in procedures of conflict resolution. If so, theoretical debate is of paramount practical relevance. Trade-offs and conflicts can be solved via a case-by-case approach, or there can be *prima-facie*-criteria for conflict resolution. Case-by-case-approaches are essentially *casuistic* and they emphasise “weighing” and “balancing”. Call this the *casuistry*-option. “Balancing” the three pillars in a *casuistic* manner might tend to convert more natural coastal areas since it might be rational in many single cases to convert them into job-generating infrastructures (harbours), industries (fisheries, mining, aquacultures, tourist destinations), settlements, and the like. A promising way to resolve and mediate conflicts might rely on spatial planning and zoning (Kenchington and Day 2011). In coastal zones, terrestrial and marine spatial planning and management systems intersect and responsibilities are often spread over several tiers of governance (see “Dealing with trade-offs and conflicts in coastal governance”). The rules of strong sustainability must be realised via integrated ecosystem-based spatial management that can effectively address transboundary impacts and includes the designation of protective areas. Call this the “spatial-resolution”-option. All national states which have coastal zones within their territory should take a *prima-facie*-commitment to do spatial planning for the minimum 10%-target (see “The 10% conservation target”).

We conclude that there are three competing concepts of sustainability which must be balanced within the overall SDG process in years to come. Most SDGs are grounded in the humanitarian concepts stemming from WCED while few SDGs directly focus on natural systems. SDG 14 with its targets belongs to this subset. In the metaphorical vision of a “healthy ocean” (target 14.2) and by the minimum of 10% target (target 14.5), a concept of strong sustainability has been implicitly presupposed. The ES approach also proposes protecting the assets of natural capitals that provide different kinds of services, including many cultural ones. In different coastal zones, the unresolved contest between different concepts of sustainability will re-emerge because coastal zones are under high pressure. If so, SDG 14 may fall prey to the many aspirations of converting more natural coastal areas. Given these premises, it makes more sense to pursue/address SDG 14 explicitly through strong sustainability than to give leeway to bargaining for weak sustainability with jobs, infrastructures, urban planning, and the like. SDG 14 should be a bulwark of the original idea of sustainability: keeping the substance of natural capitals constant over time.

## Challenges and pitfalls of a strong sustainability concept if applied to coastal zones

As outlined in “The argument for a strong sustainable SDG 14”, rules need to be in place that specify how to apply a strong sustainability concept in the context of SDG 14. The rules that need to come with such a normative framework should include (1) guidelines and management rules on how to ensure constancy of natural capital (=CNCR) and resolve *de dicto* vs. *de re* issues, (2) rules on how to minimise or reverse past failures (restoration and recovery), (3) the formulation and specification of global objectives (e.g. area-based conservation), and (4) advise on how to handle conflicts. In the application to coastal zones, the two targets 14.2 and 14.5 ought to serve as constraint functions through rules that substantiate strong sustainability. But both targets, though claimed to be robustly negotiated and reviewed in an inclusive and open process as all other SDGs and targets of the 2030 Agenda (Chasek and Wagner 2016), are much less precise in what they demand than they appear. Thus, if applied to coastal areas, the rules need to be further specified. We identify several challenges and pitfalls when applying a strong sustainability concept to coastal zones in the context of SDG 14, as discussed in the following.

### Sustainable management, protection, and restoration

A CNCR rule as implicitly formulated in target 14.2 could, for example, refer to securing ecological functions, to the conservation of species and habitat, or to the sustained supply of ecosystem services. However, target 14.2 remains factually vague in these regards. *First*, it states that marine and coastal ecosystems shall be managed sustainably, protected, and their resilience be strengthened so that “significant adverse impacts” are avoided. To determine what “significant adverse impacts” are, suitable indicators, baselines and thresholds would need to be defined. Considering the multiplicity of stressors identified for coastal regions, this would require developing a complex assessment and monitoring framework that can trace the social–ecological interactions of activities at various spatial and temporal scales and on the basis of best available information and knowledge. Sustainable management would then be reactive and adaptive to emerging “significant adverse impacts” such as coral bleaching, collapse of a fish stock or pollution above defined levels, and be combined with ecosystem-based management. A good practice example for such an approach is the management of the Great Barrier Reef Marine Park (GBRMP) in Australia. The GBRMP is subject to a wide range of drivers, including coastal development, tourism and fishing, and to recurring issues such as large-scale coral bleaching events (Hughes et al. 2017; Hughes and Kerry 2017). The Great Barrier Reef Marine Park Authority (GBRMPA) established and gradually optimised an adaptive governance system and reporting structure for assessing the effectiveness of management responses (Dobbs et al. 2011; Day and Dobbs 2013; GBRMPA 2014). However, large-scale events such as the recent 2016 and 2017 mass bleaching of corals require action both at regional (GBRMP) level and at global level in order to build resilience and foster recovery (Hughes et al. 2017).

*Second*, target 14.2 requests “restoration to achieve healthy and productive oceans”. The two main outcome aspects addressed here, “health” (see “The metaphorical vision of “healthy” ecosystems and “healthy ocean””) and “productivity”, would also need to be assessed through suitable indicators and defined thresholds to effectively describe the success of restoration measures and the status of the oceans, which should include coastal systems from our reading of the overall target. The indicator proposed by the UN’s Inter-agency Expert Group on SDG Indicators (IAEG-SDGs) to measure the achievement of target 14.2 refers to the “proportion of national exclusive economic zones managed using ecosystem-based approaches” (United Nations 2016), i.e. to measuring the spatial area subject to management actions requested. This indicator might be interpreted as one to control the CNCR rule, even though it dismisses the restoration rule and does not offer any means to monitor the outcomes of the rules.

### The 10% conservation target

Target 14.5 appears, at first glance, much more precise in its constraint function and the measuring of its outcome than target 14.2. It requests conservation status for at least 10% of coastal and marine areas each by 2020, according to our interpretation (see “The presupposition of a 10% conservation objective”), and in consistence with “national and international law and based on best available scientific information” (United Nations 2015). It is not further specified, though, where these conservation areas shall be located, whether as one large area or as several smaller conservation sites along the roughly 356,000 km of coastal zones globally, or under what conservation status. The IAEG-SDGs suggests measuring the “coverage of protected areas in relation to marine areas” as indicator for this target (United Nations 2016). But there are several challenges to this target if interpreted in the sense of a strong sustainability concept and if used as a constraint function, which are in brief: *What, where, how and why*?

The first question that needs to be answered is *what* habitats and/or species require attention, a question that is closely linked to the question of *where* (within national marine jurisdiction) to allocate the 10% conservation areas. Assuming that target 14.5 requires 10% of coastal zones to be conserved, it can be disputed whether conservation of pristine coastal areas is as relevant as the conservation of heavily modified or utilised coastal areas, or vice versa, and what outcomes each would deliver (Devillers et al. 2015; Jones and De Santo 2016). The first option might aim at conserving special or unique coastal and marine areas, while the latter would aim to avoid excessive levels of modification or use, to restore “past failures”, or to provide reference or restoration zones within intensely used coastal systems (Cinner et al. 2016).

The question of *how* relates to the conservation status and the question whether total conservation, e.g. the designation of no-take and no-use areas, guarantees better biodiversity outcomes than the establishment of managed reserves (Costello and Ballantine 2015; Edgar et al. 2014). The International Union for Conservation of Nature (IUCN) distinguishes six types of protected areas (Dudley 2008), and it needs to be answered both from a scientific and from an ethical point of view whether the 10% conservation areas should be devoted to, e.g., category Ia (“strict nature reserve”), category II (“national park”) or category VI (“protected area with sustainable use of natural resources”), or to a combination of different categories, and how these should be applied in a coastal context. This is of high relevance since conservation and human uses receive different priorities under the IUCN’s classification: while there are strict constraints on human presence in category I areas, and substantial constraints in category II areas that limit access and uses to non-extractive recreation, tourism and education, category VI establishes an overarching regime that links conservation of ecosystems and habitats to the sustainable use of natural resources and “associated cultural values and traditional natural resource management systems” (Dudley 2008: 22).

Therefore, there is urgent need for debate on how different protected area categories should be addressed within the 10%-target. There are various possibilities to relate and split conservation areas and conservation categories. We will not go into all logical possibilities here. But we claim that, if a concept of strong sustainability is to be applied, SDG 14.5 should make use of all IUCN categories, including strict regimes under categories I and II. Nevertheless, conserving 10% of coastal areas under IUCN-category I, II or VI while leaving 90% without protection would likely not be sufficient to meet the idea of strong sustainability and for ensuring CNCR (Ott 2015). Furthermore, this debate must also deal with the critical size of individual protected areas and the need for networking between sites, and it must consider the specific biogeophysical characteristics of coastal systems. With their fluid regimes and cross-boundary linkages, they have different habitat and conservation issues and thus require different approaches to conservation than terrestrial systems (Hutchings and Kenchington 2015; Kenchington 2016). Decisions over appropriate conservation areas should thus build on biophysical understanding and priorities on range, linkages and critical sites, and on vulnerabilities of species or communities, among others (Agardy et al. 2003, 2016; Devillers et al. 2015). They should also respect social and economic considerations of human dependencies and opportunities, constraints to implementation, and the desire and support for integration, especially in the context of coastal zones which, by our definition (“Introduction”), cross the interface between land and sea and by that different accustomed governance and management realms.

In this context, the *why* question needs to be answered, too, i.e. the evidence base upon which all decisions are based needs to be clear and justified. Target 14.5 requires that conservation decisions are based upon best available scientific knowledge. In our reading, this needs to include critical and science-based reflections of the target’s aims, analysis of the current “health” condition of coastal and marine areas in the respective regions and beyond, and an assessment of the effectiveness of measures and management regimes to ensure optimal conservation outcomes. There is the danger that coastal states might prioritise ease of declaration over ecological requirements under an obligation or desire to achieve a prescribed target, or decide in favour of “residual” areas (Devillers et al. 2015). Given typical histories of use and entitlement of nearshore coastal areas for subsistence, recreational and commercial fishing, cultural and aesthetic values, or nature based recreation, sport and tourism, there is also a clear need to include the humanities with respect to the domain of cultural services.

Furthermore, it has not been specified whether *each* coastal state is required to designate 10% of its coastal area to the maintenance of biodiversity and ecosystem processes, or whether this is a global target. The rules of strong sustainability would not allow realising the 10%-target, for example, only in arctic regions, even if 10% might be reached by designating areas in Greenland, Canada, Siberia, the US, and Antarctica. It requires that sufficient (representative) coastal areas are preserved and maintained in a natural state and, as far as possible, undisturbed by human activity across all bioregions. Another unanswered question is whether the 10% target applies to states with very long coastlines, as Australia or Chile, or to island states with a very large maritime space as the Philippines, in the same way as to states with much smaller ones as Singapore. The question might arise whether Chile could trade coastal conservation measures with other states that are unable to reach a meaningful 10%-target on their own territory. This, then, would feed back to the question whether strong sustainability requires an equal allocation of conservation status to all the different kinds of coastal ecosystems, and whether a certain amount of trading between different kinds of coastal areas would be acceptable so long as the global ratio of protected ecological habitats and conservation outcomes remains the same. These questions also relate to the question of substitutability within the context of CNCR, e.g. whether substituting the conservation of a beach for turtle nesting against a bird sanctuary is ecologically (or morally) appropriate.

Last but not least, there is the question of how to deal with anticipated changes of coastal areas due to sea-level rise, as projected (Church et al. 2013; Wong et al. 2014). Within such changes, and besides the various negative impacts on coastal systems, there could be many management options for designing new natural coastal areas, creating habitats for wildlife or developing more sustainable ways of doing aquacultures and tourism. Sea-level rise might also create new wetlands and entail new ways to deal with intrusion of seawater, for re-designing settlements, and more creative ways to adapt (Weston 2012: ch. 5).

Referring to our working definition of “coastal zones” (“Introduction”) which comprises transitional systems at the land–sea interface, we identify a logical issue between SDG 14 and SDG15: depending on the definition adopted, coastal areas would be addressed both through SDG 14 and through SDG 15 which aims to “protect, restore and promote sustainable use of terrestrial ecosystems” (United Nations 2015: 24). However, target 15.1^7^ does not speak of a clear and measurable conservation objective as does target 14.5. But by referencing “obligations under international agreements”, target 15.1 informs about responsibilities under the CBD’s AICHI target 11 which aims for “at least 17 per cent of terrestrial and inland water” to be conserved by 2020 (CBD 2010a). Such reading would challenge the current governance systems which, in most cases, differentiate between land and sea through lines drawn by jurisdictions or provided in legal frameworks such as UNCLOS, neglecting the transitional character and manifold interactions between the two spheres along coastal systems.

### Dealing with trade-offs and conflicts in coastal governance

When implementing the SDGs in coastal areas and setting up monitoring and accountability frameworks, interlinkages between targets 14.2 and 14.5 with others SDGs and targets of the “integrated and indivisible” 2030 Agenda (United Nations 2015) need to be taken care of to avoid negative trade-offs and ensure positive synergies. Several options are in place for navigating between constraints and mediating conflicts (see “Conceptual scheme for assessing and judging conflicts and trade-offs”). But there is the danger that policy- and decision-making neglect the requirements put forth by a strong sustainability approach if the interdependencies are not understood well enough, and if unresolved economic, political or societal issues guide decision-making. Coastal zones are complex social-ecological systems characterised by inherent multifaceted interactions and uncertainties. Coastal governance and management issues are often rather “wicked problems” with no simple dualisms between environmental conservation and socio-economic development (Kenchington et al. (2012). Thus, coherent policy making and implementation that integrates different sectors and societal actors across geographical and jurisdictional boundaries and scales is imperative for coastal governance if aiming to apply a strong sustainability approach. The State of New South Wales (NSW) in Australia made a step in such a direction by establishing an authority that brings together actors from different governmental sectors as well as non-governmental experts, the NSW Marine Estate Management Authority (MEMA 2013). MEMA assists and advises the NSW State Government for a whole-of-government approach to coordinated and evidence-based decision-making and management of what was defined as the “NSW Marine Estate” (MEMA 2013). Established only in 2013, it remains to be observed what MEMA could achieve and how well sectoral interests between, e.g., development and conservation or jurisdictional responsibilities and interests between local, State and Federal governments could be coordinated, and whether the vision of a “healthy coast and sea, managed for the greatest well-being of the community, now and into the future” was brought forward (MEMA 2013: 3).

However, different countries and regions have different socio-economic and environmental conditions, and probably rather different aspirations with regard to (sustainable) development. And they might claim the right to determine priorities based on their individual challenges and needs despite the determined universality of the 2030 Agenda and SDGs. Nations inevitably set different priorities for sustainable development depending on their individual developmental challenges, even when there is broad agreement over a strong sustainability approach. This raises the question of how, for example, global targets should be interpreted and implemented nationally, and how countries can be engaged to follow a unified approach and avoid compliance problems. SIDS, for example, strongly support and seek to implement SDG 14 (see Pacific Community 2015), and they are especially interested to apply strong sustainability for preserving the livelihoods of their people.

Another question that we need to ask in this context is whether an application of a strong sustainability concept in the implementation of targets 14.2 and 14.5 for coastal zones requires that all SDG 14 targets should follow the established normative framework, including the means of implementation (14.a–c). And if so, we could ask whether at least the other three environmentally oriented SDGs (8, 13, and 15) should follow a strong sustainability concept for a successful implementation, too.

## Conclusions

In the first section of this article, we identified the challenges facing coastal zones and coastal sustainability. We also argued that the ongoing SDG process might be regarded as an opportunity to address these challenges on different scales through SDG 14 and the targets explicitly addressing coastal areas (14.2 and 14.5). Realising this opportunity, however, requires a clear account of the underlying normative approaches, principles, and objectives. On this account, we outlined a normative framework embedded in a concept of strong sustainability (“The argument for a strong sustainable SDG 14”), arguing that the assumptions and presuppositions conceived in SDG 14 and the targets 14.2 and 14.5 refer to this concept. Since most of the SDGs refer to humanitarian aspirations, there are reasons to ground SDG 14 as one of the “environmental” SDGs in a concept of sustainability that does not allow for ongoing substitution of natural capital but provides for restoration, rehabilitation, and conservation. From our perspective, target 14.5 deserves specific attention with regard to the objective of conserving “at least 10 per cent of coastal and marine areas” (United Nations 2015: 24) and the actual implementation of such areas in coastal zones, and to the basis of decision-making. Similarly, target 14.2 raises the need for clarity on issues of sustainable management in order to avoid “significant adverse impacts” (United Nations 2015: 23) and for defining and maintaining of “healthy” coastal ecosystems. In the section on “Challenges and pitfalls of a strong sustainability concept if applied to coastal zones”, we discussed obstacles and trade-offs and provided brief examples of management consistent with implementing SDG 14 to coastal zones through a rule-based normative framework of strong sustainability.

Besides addressing specific questions about the application of the rules so that the targets 14.2 and 14.5 succeed as constraint functions, we also drew attention to conflicts arising due to the connectivity of the SDG matrix and the complexity of coastal issues. The inclusive and indivisible nature of the 2030 Agenda, purposefully introduced to capture all pressing global challenges for sustainable development, also provides potential for tensions and trade-offs due to linkages and interdependencies between (and within) the 17 SDGs and 169 targets. To resolve conflicts and avoid potential bargaining away of the environmental dimension of sustainability, coherent, integrated and adapted coastal governance is imperative. Coastal governance also should not rest on a narrow definition of coastal zones, but take due regard of the land–sea nexus of interactions, and of the various processes impacting on coastal zones, including the human dimension. There are strong indications that the pressures on coastal zones will rather increase than decrease in the future through tourism, trade and transport, increasing demand for food, energy, resources and the like, pollution and littering, and climate change.

Recognising that the term “coastal zone” or “coastal area” is insufficiently defined in the relevant documents, we have offered a working definition of coastal zones which seems appropriate for understanding and implementing SDG 14 to coastal systems. A technical definition that restricts “coastal zones” to water bodies would split land and sea apart *ex definitione* and would imply a radical belief revision of what coastal zones “are” to most people. Given our working definition of “coastal zones”, both SDG 14 (with an explicit 10% conservation target) and SDG 15 (with an implicit 17% conservation target) might be applicable for coastal areas. Such a reading would vastly challenge existing governance structures but be possibly inevitable for ensuring coherent management across the land–sea interface.

The strong sustainability framework developed in this article can only be a starting point for a comprehensive nature conservation theory for coastal and marine areas in the context of the 2030 Agenda and the SDGs. Beyond this, the hypothesis of a presupposition of strong sustainability as implicitly adopted in all environmental SDGs should be further researched. An applicable and detailed concept of strong sustainability should be developed that holds for all natural capitals addressed in the 2030 Agenda and guides the implementation process ahead.

## Footnotes

1 “By 2020, sustainably manage and protect marine and coastal ecosystems to avoid significant adverse impacts, including by strengthening their resilience, and take action for their restoration in order to achieve healthy and productive oceans” (United Nations 2015: 23).

2 “By 2020, conserve at least 10 per cent of coastal and marine areas, consistent with national and international law and based on the best available scientific information” (United Nations 2015: 24).

3 For a discussion on the challenge of reconciling the dichotomy of economic and ethical values to develop an ethical basis for sustaining coastal and ocean systems, we refer to Auster et al. (2009).

4 For reasons of brevity, we leave it open how this approach might be reconciled with discounting future events (see contributions in Hampicke and Ott 2003).

5 This is the interpretation of Norton (2013). Leopold’s principle was not meant to be a supreme moral principle but a general pragmatic maxim of how to treat land properly.

6 The ES approach should not be accused as being a Western neo-liberal economic approach which targets at the commodification of nature. ES as such is silent on property rights and governance schemes.

7 “By 2020, ensure the conservation, restoration and sustainable use of terrestrial and inland freshwater ecosystems and their services, in particular forests, wetlands, mountains and drylands, in line with obligations under international agreements.” (United Nations, 2015: 24).
